# Evaluation of the potential nephrotoxicity and mechanism in rats after long-term exposure to the traditional Tibetan medicine tsothel

**DOI:** 10.1080/13880209.2018.1543332

**Published:** 2018-12-17

**Authors:** Li Xiang, Bo Lin, Ping Wang, Yingfan Hu, Jiasi Wu, Yong Zeng, Xianli Meng

**Affiliations:** a College of Pharmacy, Chengdu University of Traditional Chinese Medicine, Chengdu, P.R. China;; b Pharmaceutical Department, The Second Affiliated Hospital of Hainan Medical University, Haikou, P.R. China

**Keywords:** Mercury, stress-related effects, detoxifying enzyme system

## Abstract

**Context:** Tsothel, a traditional Tibetan medicine, is regarded as ‘the king of essences’. Nevertheless, tsothel has aroused serious concern regarding its biosafety because its main component is HgS. Unfortunately, toxicological studies on tsothel are scarce.

**Objective:** As inorganic mercury has high affinity for the kidney, the present investigation was designed to determine the potential nephrotoxicity and mechanism of tsothel.

**Materials and methods:** Sprague-Dawley rats were orally administered different doses of tsothel (0, 66.70, 33.35 and 16.68 mg/kg) daily for 180 days, followed by the withdrawal of tsothel for 120 days. Then, the related nephrotoxicity was examined by the ICP-MS, ELISA, colorimetric, RT-PCR, HE staining, immunohistochemical staining and flow cytometry methods.

**Results:** Although tsothel administration led to a large accumulation of Hg (794.25 ± 464.30 ng/g in the 66.70 mg/kg group, 775.75 ± 307.89 ng/g in the 33.35 mg/kg group and 532.60 ± 356.77 ng/g in the 16.68 mg/kg group) in the kidney after 120 days of tsothel withdrawal, the blood CREA and BUN, urinary Kim-1, NAG, RBP and β2-MG, renal SOD, MDA, pathology, proliferation, apoptosis and cell cycle had no significant changes compared with the control group. Additionally, the high GSH content (318.87 ± 44.19 nmol/mL in the 33.35 mg/kg group) and the relative expression levels of Kim-1 (1.08 ± 0.11 in the 33.35 mg/kg group), MT-1 (1.46 ± 0.10 in the 66.70 mg/kg group, 1.61 ± 0.19 in the 33.35 mg/kg group and 1.57 ± 0.14 in the 16.68 mg/kg group) and GST-Pi (1.76 ± 0.89 in the 33.35 mg/kg group) mRNA recovered to normal after tsothel withdrawal. Interestingly, the change trend of GST-Pi gene expression was consistent with the change trend of GSH activity.

**Conclusions:** Overall, our study shows that tsothel administration did not induce overt nephrotoxicity but did have reversible stress-related effects. These results suggest that tsothel affects stress response mechanisms with the involvement of detoxifying enzyme systems. The formulation method and chemotype could play a role in the reduced toxicity potential of tsothel compared to common mercurials.

## Introduction

Traditional Tibetan medicine is an ancient and intricate holistic system of health care that utilizes complex herbal and mineral pharmaceuticals (Zuskin et al. 2008). It is a unique healing system that uses a multimodal, individualized patient approach in an attempt to bring the body, mind, and spirit into harmony (Roberti di Sarsina et al. 2011). Because of its comprehensive and flexible treatment strategies regularly bring about good treatment results, an increasing number of people around the world are interested in Tibetan medicine (Zhou et al. 2016). However, similar to some other ethnomedicines, Tibetan medicine also faces severe challenges, such as heavy metals. One reason why Tibetan medicines are forbidden in the West is that the content of heavy metals in these medicines does not meet the allowable standards of Western countries.

For example, tsothel is a notable metal-containing traditional Tibetan medicine that contains 54% mercuric sulphide (HgS). It’s named ‘བཙ་ཐལ།’ in the Tibetan language, which can be transliterated into ‘Zuo ta’ and ‘Zuo tai’ in Chinese pinyin, or ‘*Rin chin dngu chu btso bkru chin mo*’ in Latin. The name means that its special processing method looks like ‘burning into powder’ (He et al. 2006; Kan 2013). Tsothel is treated as ‘the king of essences’ in Tibetan medicine because it has many effects when used as a supplementary material to other drugs (Kan 2013). Tsothel is often used in important clinical areas, including digestive, cardiovascular, cerebrovascular, hepatic and colic diseases, and even in food poisoning, hypertension and leprosy (Yutuo 1987; Luo 2004; Kan 2013). Tsothel is believed to be able to enrich the blood and promote blood flow (Huang et al. 2013). Additionally, modern pharmacological studies have indicated that tsothel exhibits many biological effects, including sedation, hypnosis, antipyresis, anti-inflammation, enhancing immunity, anticonvulsion and bacteriostasis activity, as well as prolonging the life of female fruit flies (Zeng et al. 2005; Jiang et al. 2009; Chen et al. 2011). Many commercially available formulations contain tsothel, such as ‘Qishiwei Zhenzhu Wan’, ‘Renqing Chang Jue’, ‘Renqing Mang Jue’, ‘Zuo Zhu Da Xi’ and ‘Zuota Dezi Ma’ (Zhu 2006). Because of its long history and excellent performance, tsothel has been recognized as part of Chinese Intangible Cultural Heritage.

However, as one of the top secrets of Tibetan medicine, tsothel is well protected. Therefore, pharmacological and toxicological studies on tsothel are very scarce (Li et al. 2016). Nevertheless, it is well known that tsothel is made from mercury by a special Tibetan processing protocol (Suo 2007; Duo and Chu 2013). However, mercury is undoubtedly a toxic element (Mahboob et al. 2001; Llobet et al. 2003; Sener et al. 2003; Liu et al. 2008; Carocci et al. 2014). Tsothel also contains the ash of ‘eight heavy metals’ (mercury, gold, silver, copper, brass, iron, lead. and tin) as well as ‘eight mineral substances’ (native copper, gold ore, silver ore, magnetite, ettringite, orpiment, realgar and red mica) (Zhu 2006). Therefore, the biosafety of tsothel has raised serious doubts (Yang 2004; Dou 2005; Li et al. 2010; Chen et al. 2011). To date, studies have shown that mercury accumulates in some organs, especially in the kidney, after the long-term and large-scale use of tsothel (Yang 2004; Li et al. 2010; Li et al. 2016). Furthermore, these results were consistent with the finding that the kidney is the main target organ of the transport, accumulation and toxicity of inorganic mercury, including mercuric chloride and HgS (Zalups and Lash 1994; Tchounwou et al. 2003; Liu et al. 2008; Jan et al. 2011; Lu et al. 2011; Shi et al. 2011). Unfortunately, the potential toxicity and mechanism of tsothel, especially its nephrotoxicity, have not been reported.

Consequently, more detailed studies are needed to understand the risk associated with the use of HgS-containing medicines (Mao and Desai 2009). Our present study, which aimed at the accumulation of mercury and the potential toxic effects and mechanism of tsothel in rats, focussed on injury to the kidney, a major target organ for chronic inorganic mercurial poisoning. The results showed that tsothel can cause potentially reversible injury to the kidney. The mechanism, at least in part, is probably closely associated with the stress response and detoxification enabled by the detoxifying enzyme systems.

## Materials and methods

### Chemicals

Tsothel (54% HgS) obtained from the Tibet Autonomous Region Tibetan medicine factory was used in the present study. Element analysis of tsothel by X-ray fluorescence shows that it is composed of Hg (52.909%), S (24.992%), O (4.401%), Fe (1.801%), Cu (0.411%), Si (0.294%), Zn (0.291%), Ag (0.222%), Sr (0.206%), Mg (0.178%), Ca (0.153%), K (0.137%), Al (0.113%), Cr (0.002%) and Rb (0.100%). The sodium carboxymethyl cellulose (CMC-Na) used for preparing tsothel suspensions was purchased from Sigma-Aldrich (St. Louis, MO, USA). All other chemicals and solvents used were of analytical grade.

### Animals

Apparently healthy Sprague-Dawley rats (*N* = 184, 2 2 ∼ 26 days, 6 0 ∼ 80 g), half male and half female, were purchased from Chengdu Dashuo Bio-Technique Co. Ltd. (Chengdu, China). Prior to the experiment, all rats were kept in the laboratory conditions under standard management for 7 days for acclimatization. The animal house has controlled environmental conditions, with a temperature and relative humidity of approximately 22 ± 2 °C and 50 ± 8%, respectively, and artificial lighting alternating on a 12 h light-dark cycle. The rats were given a standard balanced diet and distilled water *ad libitum* throughout the experiment. The distilled water was preferred to avoid any trace amount of impurity present in the tap water. All experimental procedures were carried out according to institutional guidelines for the care and use of laboratory animals at Chengdu University of Traditional Chinese Medicine, Chengdu, China (NO. TCM-2009-315), and the National Institutes of Health Guide for the Care and Use of Laboratory Animals (8th Edition, 2012). The permission number from the ethics committee was 20130918021. In addition, all efforts were made to minimize the number of animals used and their suffering.

### Experimental design

The tsothel doses (66.70, 33.35 and 16.68 mg/kg) selected were 100-, 50- and 25-fold greater than the clinical dose (0.667 mg/kg), respectively. Rats were grouped evenly by weight. One was the control group, and the other three were the high-, median- and low-dose tsothel groups, respectively. During the experiment, the control group was given 0.5% CMC-Na suspension intragastrically (i.g.), while the high-, median- and low-dose tsothel groups were injected i.g. with 66.70, 33.35 and 16.68 mg/kg tsothel, respectively. All oral administrations were given once a day for 180 days with 1 day of drug withdrawal every week and were weighed weekly to adjust the dose. Additionally, the rats were observed daily for gross symptoms and signs of toxicity and mortality throughout the experiment.

Sample size was determined by power analysis using preliminary data obtained in our laboratory with the following assumptions: α of 0.05 (two-tailed) and power of 90%. Forty rats were used after 90, 135 or 180 days of tsothel administration and 30 days of tsothel withdrawal, whereas 24 rats were used after 120 days of tsothel withdrawal. A day before being sacrificed, the rats were transferred to metabolic cages individually with free access to water to collect the nocturnal urine for 12 h. Urine samples were centrifuged at 3500 rpm for 10 min at 4 °C. Then, the supernatants were collected, snap-frozen in liquid nitrogen and subsequently stored at –80 °C for further analysis. Ten rats (half male and half female) in each group were randomly selected and euthanized after 90, 135 and 180 days of medication and 30 days of drug withdrawal. Similarly, the remaining 6 rats (half male and half female) in each group were sacrificed after 120 days of tsothel withdrawal. During each sampling, the selected rats were euthanized by drawing blood from the abdominal aorta under mild urethane anaesthesia. Blood was collected from the abdominal aorta and divided into two parts. One sample was allowed to sit undisturbed for 1 h, and then the serum was separated by centrifugation at 3500 rpm for 10 min at 4 °C, while the other sample was transferred into heparinized tubes and snap-frozen in liquid nitrogen directly. Subsequently, the serum and blood were transferred and stored at –80 °C for further analysis. Kidneys were removed immediately after drawing blood, washed with saline, blotted dry on filter paper, weighed, and cut into 5 parts. One section was preserved in cold phosphate-buffered saline (PBS), and another section preserved in 4% paraformaldehyde, whereas the remaining 3 renal samples were put into cryotubes, snap-frozen in liquid nitrogen and then stored at –80 °C. All of the renal samples, preserved in different ways, were used for further analysis.

### Determination of Hg in blood and kidney

The total Hg concentrations in the blood and kidney were quantified by inductively coupled plasma mass spectrometry (ICP-MS). Briefly, blood and renal tissue samples were added to the microwave digestion instrument and allowed to completely soak in 5 mL of nitric acid overnight before digestion. Subsequently, the samples were digested in 5 mL of HNO_3_ at 120 °C, 150 °C and 180 °C successively for 10, 10 and 20 min, respectively. Then, the digestion solutions were completely transferred to a volumetric flask, supplemented with 200 μL of gold standard solution, and brought to 50 mL with distilled water. Finally, the Hg concentrations in the blood and kidney were detected by the ICP-MS method with a 7700 series mercury-measuring instrument. The blood Hg levels were expressed as nanograms per millilitre of blood, while the levels of renal Hg were expressed as nanograms per gram of tissue.

### Blood, urine and kidney biochemical analysis

Creatinine (CREA) and BUN concentrations in serum; Kim-1, β2-MG and RBP concentrations in urine; and MDA, Kim-1, MT and GSH contents in renal tissue were determined by an enzyme-linked-immunosorbent assay (ELISA) kit from Beijing Ya An Da Biotechnology Co. Ltd. (Beijing, China). NAG activity in urine and SOD and GSH-Px activities in renal tissue were detected using a colourimetric method. A NAG assay kit was obtained from Sigma-Aldrich (St. Louis, MO, USA). SOD and GSH-Px assay kits were purchased from Beyotime Biotechnology Co. Ltd. (Wuhan, China). All of the detection procedures were strictly performed in accordance with the instructions.

### RNA isolation and real-time RT-PCR analysis

Approximately 6 0 ∼ 100 mg of kidney tissue, previously cryopreserved, was homogenized in 1 mL of TRIzol reagent (Invitrogen, Carlsbad, CA, USA), and total RNA was extracted according to the manufacturer’s instructions, followed by purification with RNeasy kits (Qiagen, Valencia, CA, USA). The quality of RNA was determined by the 260/280 ratios. The total purified RNA was reverse transcribed with MMLV reverse transcriptase and oligo-dT primers. Power SYBR Green Master Mix (Applied Biosystems, Foster City, CA, USA) was used for real-time reverse-transcriptase polymerase chain reaction (RT-PCR) analysis. The primers were designed by Primer Express software (Applied Biosystems, Foster City, CA, USA) and are listed below (Table 1). Differences in gene expression were calculated using cycle threshold (Ct) values, which were first normalized against β-actin levels in the same sample and expressed as relative transcript levels, setting the controls as 100%.

**Table 1.** Sequences of the primers.

Gene	Gene bank number	Forward	Reverse
β-actin	NM_031144	GGCCAACCGTGAAAAGATGA	GCCTGGATGGCTACGTACATG
Kim-1	NM_173149	TGGCACTGTGACATCCTCAGA	GCAACGGACATGCCAACATA
MT-1	NM_138826	TGTGCCTGAAGTGACGAACAG	TTCACATGCTCGGTAGAAAACG
GST-Pi	X02904	GGGTCGCTCTTTAGGGCTTTA	GCAGGGCCTTCACATAGTCATC

### Histopathology and immunohistochemistry

A portion of the kidney was fixed in 4% paraformaldehyde for at least 48 h at room temperature prior to processing. Subsequently, the fixed tissues were ethanol-dehydrated, paraffin-embedded, sectioned at 5 μm and stained with regular haematoxylin and eosin (H&E) for histological analysis. Pathological assessments were performed in a blind fashion. The incidence of renal lesions was determined by histologic analysis of a longitudinal midline section of a kidney from each rat.

To evaluate renal cell proliferation activity, immunohistochemical examination of Ki67 protein was performed. The renal tissue sections, which had been fixed in 4% paraformaldehyde, ethanol-dehydrated, paraffin-embedded and sectioned at 5 μm, were mounted on glass slides. To block endogenous peroxidase activity, sections were incubated with 3% H_2_O_2_ for 10 min. The sections were incubated overnight at 4 °C with a rat monoclonal antibody against Ki67 (eBioscience), then were incubated with a biotinylated secondary antibody (Vector, Burlingame, CA) for 20 min at 37 °C, and finally were stained with diaminobenzidine (DAB). The sections were counterstained with haematoxylin for 3 min. For negative staining, the primary antibody was omitted from the incubation step. The positive area of Ki67 expression was detected by an IMS Image Analysis system (JRDUN Biotechnology (Shanghai) Co. Ltd, China).

### Apoptosis and cell cycle analysis

Renal cells from the kidney tissues preserved in cold PBS were immediately isolated by a two-step collagenase perfusion technique (Li et al. 2010). Cell apoptosis was detected by flow cytometry (FACS Calibur, BD, Franklin Lakes, NJ, USA). The levels of apoptosis were measured according to the annexin V-FITC–PI double-staining method (Li et al. 2007) and were expressed as the rate of apoptosis, which was shown in the FCM squares. The cell cycle distribution was assayed by determining the DNA contents. Cells were fixed in 70% ethanol overnight at 4 °C and resuspended in a staining solution containing RNase A and propidium iodide (PI) for 20 min. After washing, the DNA content was determined by flow cytometry and analyzed with CELLQUEST software. Both apoptosis and cell cycle analysis performed according to the Kit manufacturer’s instructions from Nanjing Keygen Biotech. Co. Ltd. (Nanjing, China).

### Statistical analysis

All data are expressed as the means ± SD. Statistical analysis of data was performed using SPSS17.0 software. For comparisons among three or more groups, data were analyzed using one-way analysis of variance (ANOVA) followed by least significant difference (LSD) analysis and Tamhane’s T2 test. Normality tests and variance homogeneity tests were used before ANOVA. Assuming that the data conformed to a normal distribution, LSD analysis was used with the constant variances in the data, or Tamhane’s T2 test was used with the unequal variances in the data. The Kruskal-Wallis rank test was used to assess the data that did not follow a normal distribution. Differences were considered significant when *p* < 0.05.

## Results

### Clinical manifestations

All rats in the tsothel and control groups were healthy and active. The clinical symptoms were closely monitored during the 300-day experimental period. Additionally, the spontaneous activities, grooming and food/water intake of all rats were normal.

### Blood and renal mercury accumulation

**Figure 1.**
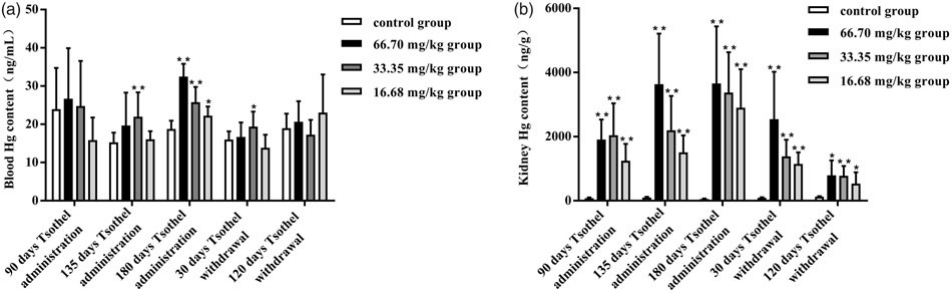
Hg concentrations in the blood (A) and kidney (B) (*N* = 184). Rats were orally administered different doses of tsothel (66.70, 33.35 and 16.68 mg/kg) daily for 90, 135 and 180 days and subjected to tsothel withdrawal for 30 and 120 days. Blood and tissues were digested in HNO_3_, and then the Hg contents were determined by inductively coupled plasma mass spectrometry (ICP-MS). Data represent the mean ± SD. *Significantly different from the control, *p* < 0.05, **Very significantly different from the control, *p* < 0.01.

The Hg concentrations in the blood and kidney after tsothel exposure are shown in Figures 1A and B, respectively. The blood Hg concentration in tsothel group was shown to not be significantly different from that in the control group after 90 days of medication. In addition, compared with those in the control group, the blood Hg concentrations in the 66.70 and 16.68 mg/kg groups were slightly higher, while the blood Hg concentrations in the 33.35 mg/kg group were significantly increased (*p* < 0.01) after 135 days of medication. However, compared with those in the control group, the blood Hg concentrations all tsothel groups increased significantly and in the dose-dependent manner (*p* < 0.01, *p* < 0.01 and *p* < 0.05) after 180 days of medication. The blood Hg levels in the 33.35 mg/kg group were still relatively high after 30 days of drug withdrawal. Eventually, compared with those in the control group, the blood Hg levels in all groups treated with tsothel returned to normal after 120 days of drug withdrawal.

Compared to the kidney Hg concentrations in the control group, the Hg concentrations in the kidneys of all the tsothel groups increased significantly and in the dose-dependent and time-dependent manner by 12-fold after 90, 135 and 180 days of medication (*p* < 0.001). These results are consistent with the finding that a large amount of Hg accumulated in the kidney after long-term oral administration of tsothel (Yang 2004; Li et al. 2010, 2014; Zhang et al. 2012). Compared with those in the control group, the kidney Hg concentrations observed in the 66.70, 33.35 and 16.68 mg/kg groups were increased by 25-, 27- and 16-fold, respectively, after 90 days of medication; 37-, 22- and 15-fold, respectively, after 135 days of medication; 70-, 65- and 56-fold, respectively, after 180 days of medication; 27-, 15- and 12-fold, respectively, after 30 days of drug withdrawal; and 6-, 6- and 4-fold, respectively, after 120 days of drug withdrawal. Overall, the renal Hg levels in the 66.70, 33.35 and 16.68 mg/kg tsothel groups decreased by 30%, 59% and 60%, respectively, but still accumulated significantly after 30 days of drug withdrawal. However, compared with the renal Hg contents after 180 days of medication, the renal Hg levels in the 66.70, 33.35 and 16.68 mg/kg tsothel groups declined by 78%, 77% and 82%, respectively; the renal levels of Hg after 120 days of drug withdrawal still essentially indicated accumulation.

### Blood, urine and kidney biochemistry

**Figure 2.**
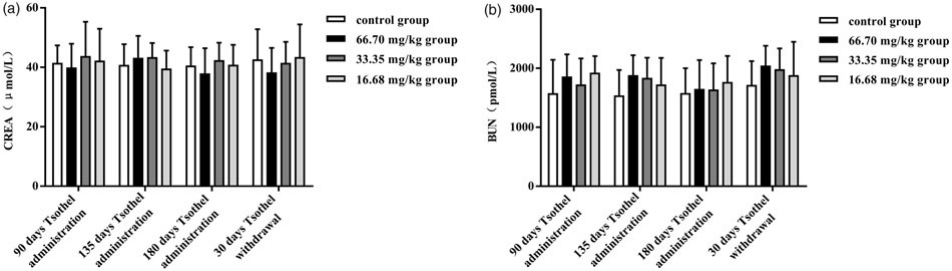
CREA (A) and BUN (B) levels (*N* = 160). Rats were orally administered different doses of tsothel (66.70, 33.35 and 16.68 mg/kg) daily for 90, 135 and 180 days and subjected to tsothel withdrawal for 30 days. Blood was collected at the termination of each phase of this experiment for analysis by enzyme-linked immunosorbent assay (ELISA). Data represent the mean ± SD. *Significantly different from the control, *p* < 0.05, **Very significantly different from the control, *p* < 0.01.

Two major biomarkers of renal damage, CREA and BUN, were assayed, as they may indicate a reduction in renal function. As illustrated in Figures 2A and B, no significant elevation in CREA and BUN was observed in any tsothel groups throughout the experiment, suggesting that CREA and BUN were not sensitive enough to detect chronic tsothel-induced nephrotoxicity.

**Figure 3.**
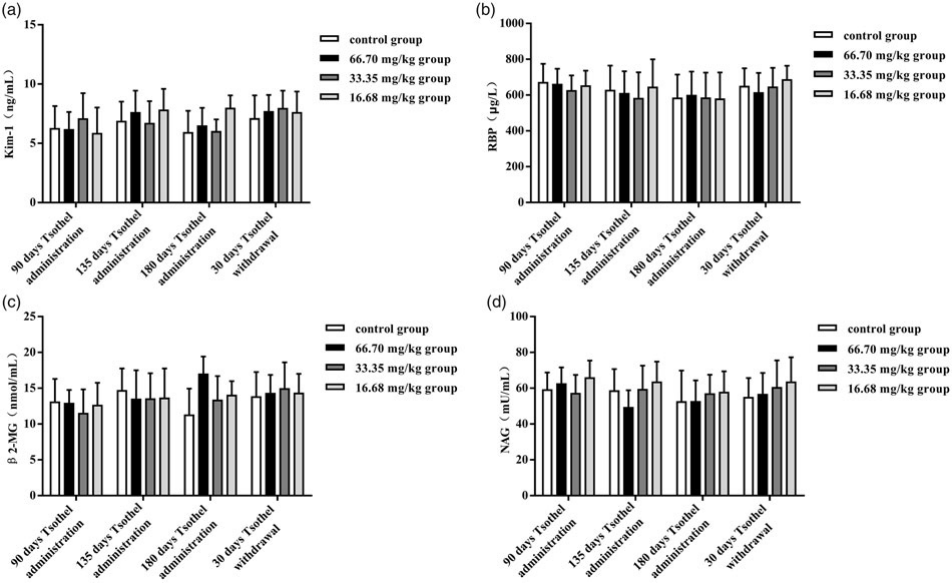
Urinary Kim-1(A), RBP (B), and β2-MG (C) contents and NAG (D) activity (*N* = 160). Rats were orally administered different doses of tsothel (66.70, 33.35 and 16.68 mg/kg) daily for 90, 135 and 180 days and subjected to tsothel withdrawal for 30 days. Urine was collected the day before the termination of each phase for analysis by enzyme-linked immunosorbent assay (ELISA). Data represent the mean ± SD. *Significantly different from the control, *p* < 0.05, **Very significantly different from control, *p* < 0.01.

Therefore, more sensitive biomarkers of early renal injury were assessed. The contents of Kim-1, RBP and β2-MG and the activity of NAG in urine after exposure to different doses of tsothel are shown in Figures 3A–D, respectively. However, there were no significant differences in urinary Kim-1, RBP, β2-MG and NAG among all groups after 90, 135 and 180 days of medication and 30 days of drug withdrawal, suggesting that tsothel did not induced obvious early renal injury.

**Figure 4.**
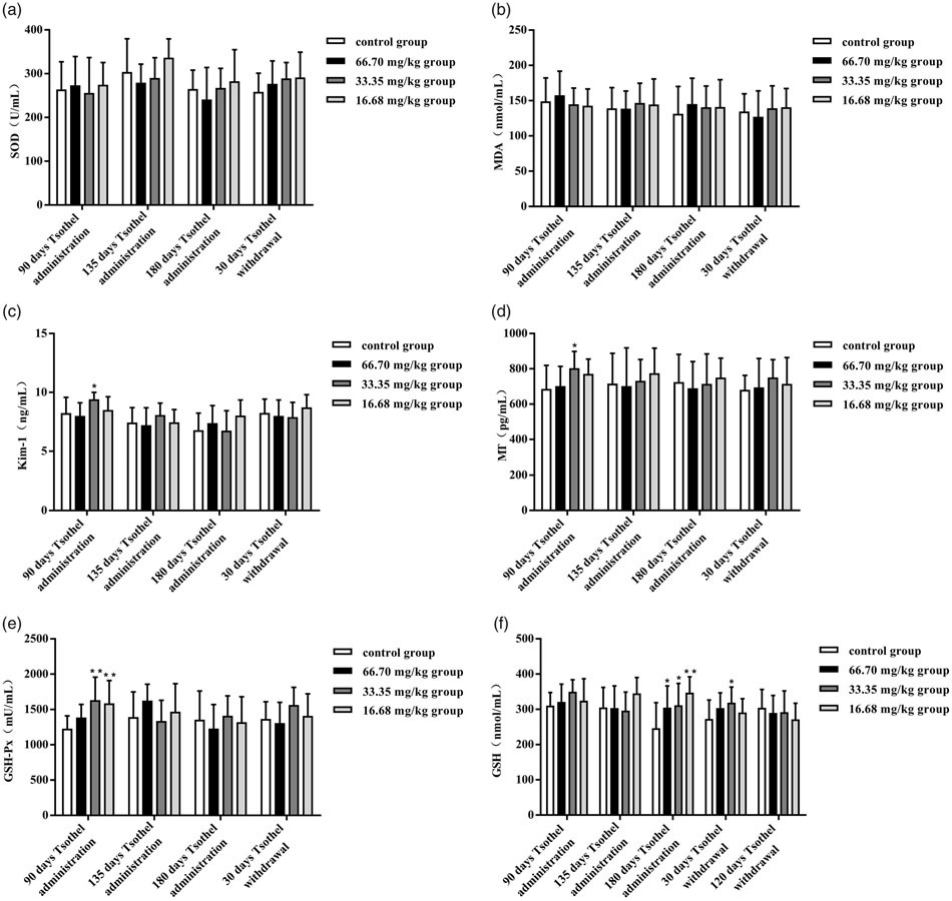
SOD (A) and GSH-Px (E) activities and MDA (B), Kim-1(C), MT (D), and GSH (F) contents in the kidney (*N* = 184). Rats were orally administered different doses of tsothel (66.70, 33.35 and 16.68 mg/kg) daily for 90, 135 and 180 days and subjected tsothel withdrawal for 30 days. Renal tissues were collected at the termination of each phase of the experiment for analysis by enzyme-linked immunosorbent assay (ELISA). Data represent the mean ± SD. *Significantly different from the control, *p* < 0.05, **Very significantly different from the control, *p* < 0.01.

The activity of SOD and the content of MDA in the kidney after exposure to different doses of tsothel are shown in Figures 4A and B, respectively, and the renal contents of Kim-1, MT and GSH and the activity of GSH-Px are shown in Figures 4C–F, respectively. There were no significant differences in SOD or MDA between any of the groups during the experiment. In addition, compared with the control group, the 33.35 mg/kg tsothel led to an increase (*p <* 0.05) in Kim-1 and MT levels after 90 days of medication. Unexpectedly, the contents of Kim-1 and MT in the 33.35 mg/kg group were very close to those in the control group after 135 and 180 days of medication and 30 days of drug withdrawal. Similarly, compared with that in the control group, the activity of GSH-Px in the 33.35 and 16.68 mg/kg tsothel groups increased significantly (*p* < 0.01) after 90 days of medication but showed no difference from the control group after 135 and 180 days of medication and 30 days of drug withdrawal. Furthermore, all the tsothel groups had significantly increased GSH content (*p* < 0.05, *p* < 0.05 and *p* < 0.01) compared with that in the control group after 180 days of medication. Unexpectedly, the GSH content in the 33.35 mg/kg group was still relatively high (*p* < 0.05) after 30 days of drug withdrawal. However, the GSH levels in the 33.35 mg/kg tsothel group eventually returned to normal after 120 days of drug withdrawal.

### Expression of genes related to renal toxicity

**Figure 5.**
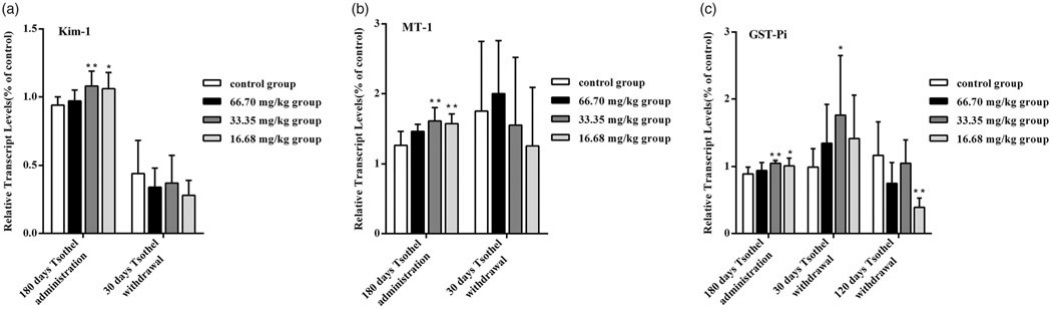
Expression of Kim-1 (A), MT-1(B), and GST-Pi (C) mRNA in the kidney (*N* = 184). Rats were orally administered different doses of tsothel (66.70, 33.35 and 16.68 mg/kg) daily for 90, 135 and 180 days and subjected to tsothel withdrawal for 30 and 120 days, and then total RNA was extracted, purified, and subjected to real-time reverse-transcriptase polymerase chain reaction (RT-PCR) analysis. Data represent the mean ± SD. *Significantly different from the control, *p* < 0.05, **Very significantly different from the control, *p* < 0.01.

The expression levels of Kim-1, MT-1 and GST-Pi mRNA in the kidney after exposure to different doses of tsothel are shown in Figures 5A–C, respectively. The expression levels of renal toxicity-related genes were determined as sensitive biomarkers for Hg-induced nephrotoxicity. Kim-1 has recently been identified as a sensitive biomarker for kidney injury (Bonventre 2008). Compared with those in the control group, after 180 days of medication, the expression levels of Kim-1 mRNA in the 33.35 and 16.68 mg/kg tsothel groups were increased significantly (*p* < 0.01 and *p* < 0.05), but those in the 66.70 mg/kg tsothel group were unchanged. The Kim-1 mRNA expression levels measured in the 33.35 and 16.68 mg/kg tsothel groups returned to normal after 30 days of drug withdrawal. The induction of MT is widely used as a biomarker for metal-induced toxicity (Klaassen et al. 1999). In this study, the expression levels of MT-1 mRNA changed significantly (*p* < 0.01) in the 33.35 and 16.68 mg/kg tsothel groups after 180 days of medication but returned to normal levels after 30 days of drug withdrawal. Moreover, glutathione *S*-transferase (GST) plays an important detoxification role through the consumption of endogenous glutathione by *S*-conjugate formation to prevent metal-induced oxidative stress. Compared with those in the control group, the expression levels of GST-Pi mRNA in 33.35 and 16.68 mg/kg tsothel groups were increased significantly (*p* < 0.01 and *p* < 0.05) after 180 days of medication, whereas those in the 66.70 mg/kg tsothel group were unchanged. Unexpectedly, the expression of GST-Pi mRNA in the 33.35 mg/kg group was still relatively high (*p* < 0.05) after 30 days of drug withdrawal. Compared with that in the control group, the expression of GST-Pi mRNA in 33.35 mg/kg tsothel group eventually returned to normal after 120 days of drug withdrawal.

### Renal pathology and immunohistochemistry

**Figure 6.**
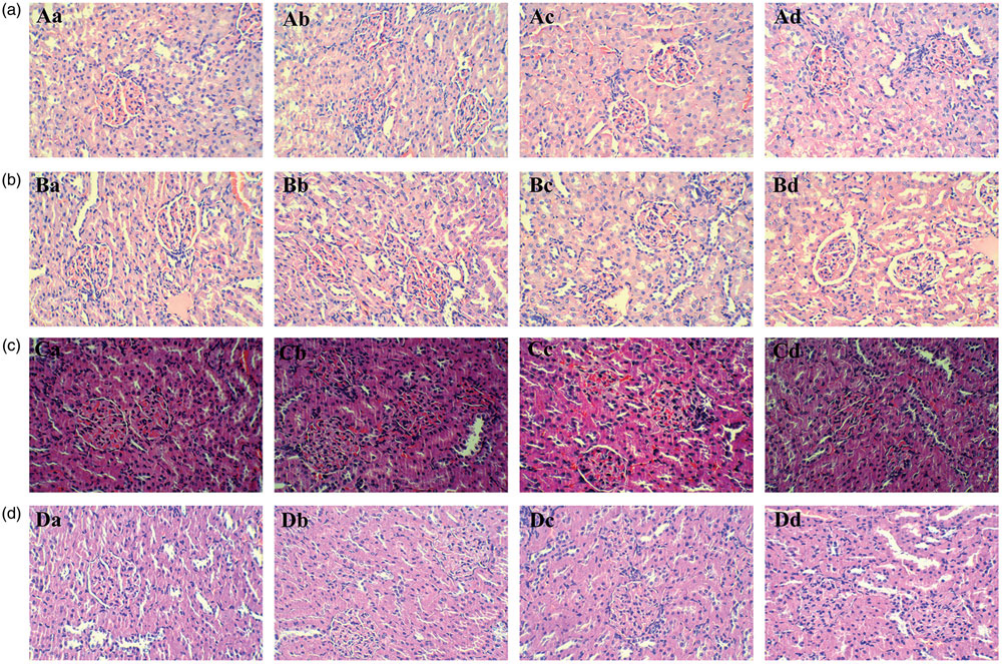
Representative histopathology images of the kidney from the rats exposed to different doses of tsothel (66.70, 33.35 and 16.68 mg/kg) after 90, 135 and 180 days of administration and 30 days of drug withdrawal (*N* = 160). (A) Representative histopathology of the kidney after 90 days of administration. (B) Representative histopathology of the kidney after 135 days of administration. (C) Representative histopathology of the kidney after 180 days of administration. (D) Representative histopathology of the kidney after 30 days of drug withdrawal. Tissues were fixed in 4% paraformaldehyde and stained with haematoxylin and eosin. H&E stain, ×200 magnification. a: Control group; b: 66.70 mg/kg group of tsothel; c: 33.35 mg/kg group of tsothel; d: 16.68 mg/kg group of tsothel.

Representative photomicrographs of the morphological changes in the renal tissue throughout the whole experiment are presented in Figure 6. Generally, pathological examination of the kidneys in all groups showed normal morphology of the renal parenchyma with well-defined glomeruli and tubules with non-significant changes. The renal capsule was complete. No obvious connective tissue hyperplasia or inflammatory exudation was observed. No vascular proliferation or atrophic fibrosis, degeneration and necrosis were observed in the glomerulus of the renal cortex. In addition, no cloudy, glassy degeneration and necrosis swelling degeneration were observed in the convoluted tubule, and no hyperaemia and inflammatory cell infiltration were observed in the mesenchyme.

**Figure 7.**
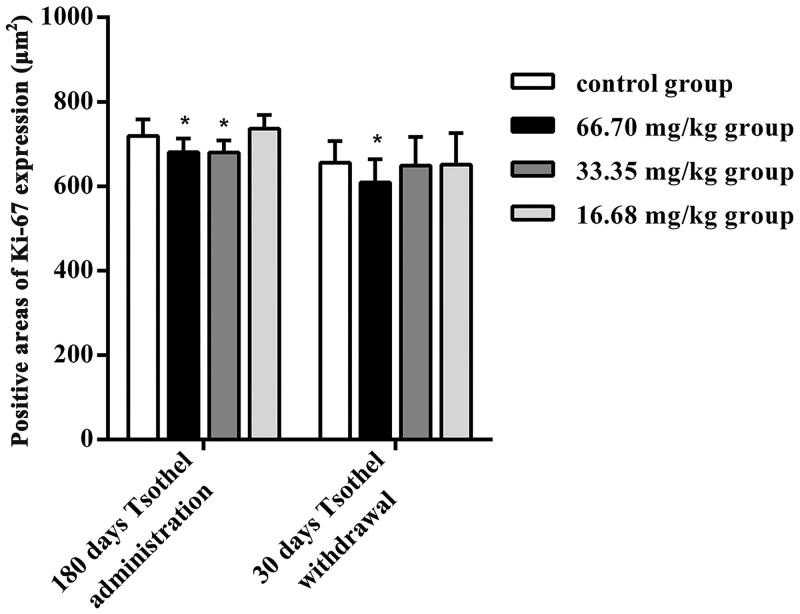
The positive areas of Ki-67 expression in rats exposed to different doses of tsothel (66.70, 33.35 and 16.68 mg/kg) after 180 days of administration and 30 days of drug withdrawal (*N* = 160).

**Figure 8.**
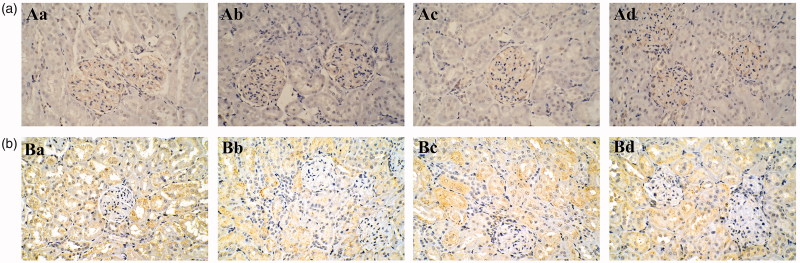
Representative Ki-67 immunohistochemistry of the kidney from rats exposed to different doses of tsothel (66.70, 33.35 and 16.68 mg/kg) after 180 days of administration and 30 days of drug withdrawal (*N* = 160). (A) Representative Ki-67 immunohistochemistry of the kidney after 180 days of administration. (B) Representative Ki-67 immunohistochemistry of the kidney after 30 days of drug withdrawal. Immunoperoxidase staining, ×200 magnification. a: Control group; b: 66.70 mg/kg tsothel group; c: 33.35 mg/kg tsothel group; d: 16.68 mg/kg tsothel group.

Immunohistochemical staining for Ki67 was performed to observe the proliferative activity of kidney cells, as shown in Figures 7 and 8. The positive immunoperoxidase staining for Ki67 in 66.70 and 33.35 mg/kg groups of tsothel was less than that in the control group after 180 days of medication. In addition, the expression (positive area) of Ki67 was still low after 30 days of tsothel withdrawal, indicating that the proliferative activity of kidney cells may be affected by tsothel.

### Renal apoptosis and cell cycle

**Figure 9.**
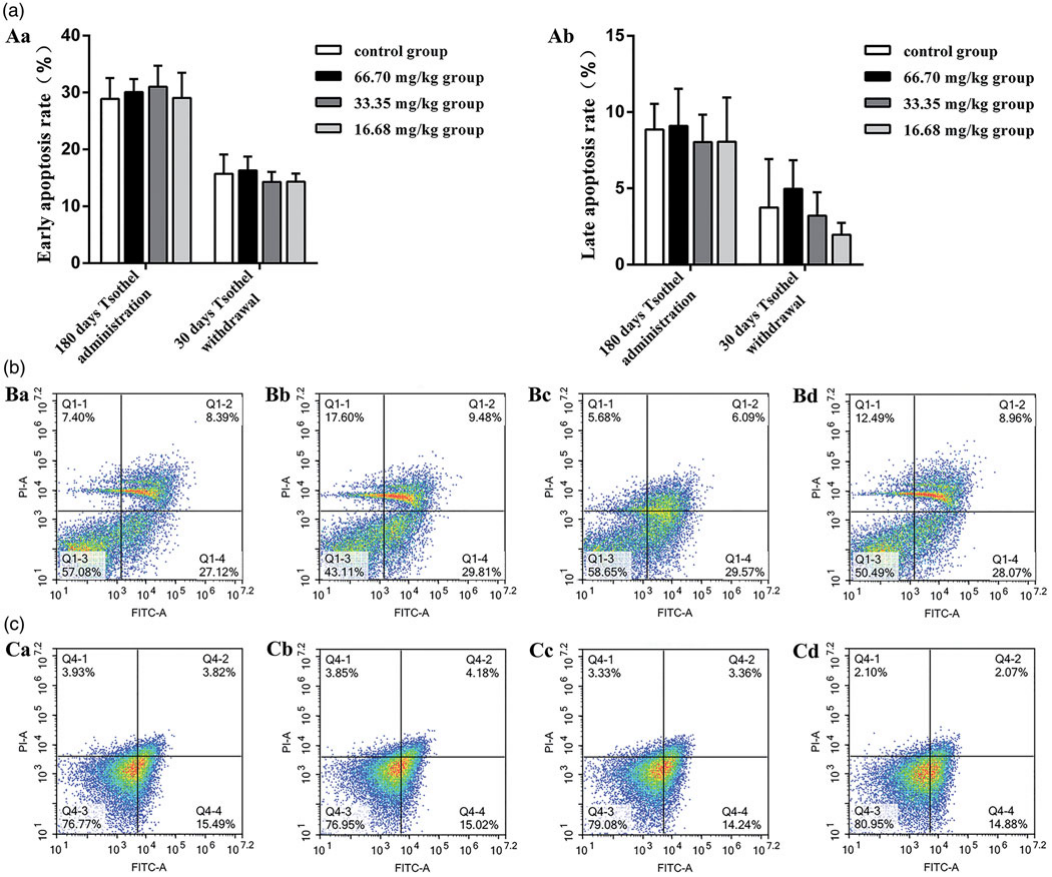
Renal cell apoptosis (*N* = 160). (A) Apoptosis rate. Aa represents the early apoptosis rate, and Ab represents the late apoptosis rate. (B) Representative apoptosis flow cytometry diagrams of renal cells isolated from kidney tissue after 180 days of administration and analyzed by flow cytometry. (C) Representative apoptosis flow cytometry diagrams of renal cells isolated from kidney tissue after 30 days of drug withdrawal and analyzed by flow cytometry. Viable cells are in the lower left quadrant, early apoptotic cells are in the lower right quadrant, late apoptotic cells are in the upper right quadrant, and non-viable necrotic cells are in the upper left quadrant. Ba and Ca: Control group; Bb and Cb: 66.70 mg/kg tsothel group; Bc and Cc: 33.35 mg/kg tsothel group; Bd and Cd: 16.68 mg/kg tsothel group.

**Figure 10.**
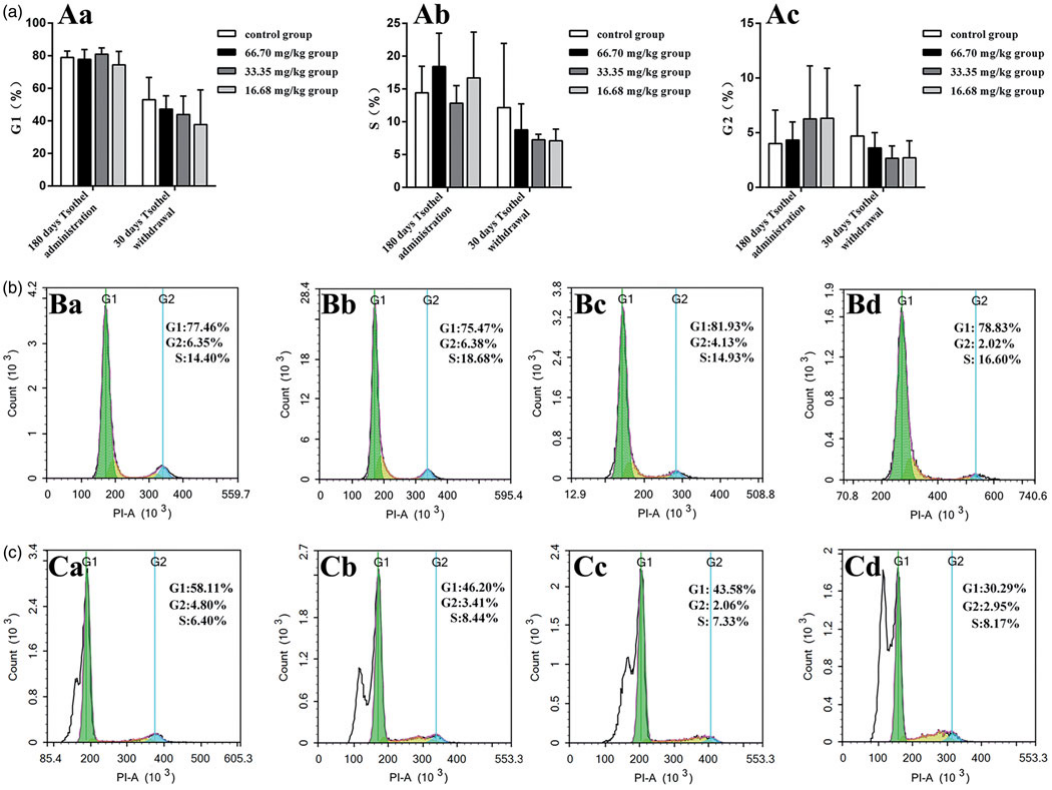
Cell cycle distribution of renal cells (*N* = 160). (A) Rates of different cell cycle phases. Aa represents the rate of G1 phase, Ab represents the rate of S phase and Ac represents the rate of G2 phase. (B) Representative cell cycle distribution diagrams of renal cells isolated from kidney tissue of rats after 180 days of administration and analyzed by flow cytometry. (C) Representative cell cycle distribution diagrams of renal cells isolated from the kidney tissue of rats after 30 days of drug withdrawal and analyzed by flow cytometry. Ba and Ca: Control group; Bb and Cb: 66.70 mg/kg tsothel group; Bc and Cc: 33.35 mg/kg tsothel group; Bd and Cd: 16.68 mg/kg tsothel group.

Representative graphs of apoptosis and the cell cycle in the renal cells throughout the whole experiment are shown in Figures 9 and 10, respectively. In the experiments, the apoptosis indicator annexin V-FITC was not markedly altered with time. The early and late apoptosis rates in all tsothel groups did not differ from those in the control group after 180 days of medication and 30 days of drug withdrawal. Additionally, cell cycle analysis indicated that there was no significant cell cycle arrest in any of the tsothel groups compared with the control group after 180 days of medication and 30 days of drug withdrawal.

## Discussion

Tsothel plays an important role in traditional Tibetan medicine. Mercury, the main ingredient of tsothel, is regarded as the main source of heavy metals in this medicine. The mercury found in traditional medicines has justifiably raised concern among the public (Ernst 2002; Cooper et al. 2007; Saper et al. 2008). The kidney is considered the major target organ for inorganic mercury toxicity in humans and experimental animals, especially after long-term administration (Liang et al. 2005, 2009; Zhou et al. 2009). Therefore, the objectives of this study were to assess the accumulation of mercury rather than trace amounts of other heavy metals and to elucidate the underlying nephrotoxicity of tsothel after long-term and large-scale oral administration.

First, high Hg concentrations in the kidney were observed, which agrees with the results of some previous studies (Yang 2004; Li et al. 2010, 2014; Zhang et al. 2012). As the most important excretory organ, the kidney has the highest blood flow volume and takes part in the elimination of exogenous material. Moreover, it is the main target organ for inorganic mercury transport, accumulation and toxicity (Zalups and Lash 1994; Tchounwou et al. 2003; Liu et al. 2008; Jan et al. 2011). The accumulation of Hg in the kidney may be explained by the high affinity between Hg and sulfhydryl (SH) groups (Bach and Weibel 1976), as many SH-containing proteins and enzymes are present in the kidney.

The unchanged CREA and BUN levels observed in our study indicate that long-term administration of tsothel does not cause an obvious reduction in renal function. CREA and BUN are two routinely used biomarkers for renal damage in preclinical and clinical diagnosis, and elevated CREA and BUN levels provide indication of the degree of renal injury.

Our results showed that compared with those in the control group, the Kim-1, NAG, β2-MG and RBP levels in each tsothel group did not significantly change throughout the experiment. As a non-invasive indicator of renal proximal tube injury, urinary Kim-1 is regarded as an ideal biomarker for detecting early-phase renal injury (Ichimura et al. 1998; Tang and Zou 2006; Xu and Tang 2010; AbdulHameed et al. 2016). Urinary Kim-1 can rapidly, sensitively, specifically, and stably reflect the injury in various kidney diseases. RBP is a low-molecular-weight protein that cannot pass through the glomerular basement membrane. Normally, little RBP is discharged into the urine, while the discharge amount of RBP is increased when the proximal convoluted tubule is injured (Xiong et al. 2010). β2-MG is regarded as a sensitive and specific biomarker for the injured reabsorption function of the renal tubules (Dieterle et al. 2010; Xu and Tang 2010). Generally, only 0.3% β2-MG appears in the urine. When the tubular reabsorption function is slightly damaged, the reabsorption of β2-MG is weakened, and thus, β2-MG will be released into the urine. NAG is widely distributed in various organs, such as the kidney. In general, it is not easy for the glomerulus to filtrate. However, when the kidney is damaged by nephritis, heavy metal or drugs, a large amount of NAG can be excreted into urine, and this effect appears earlier than other indicators of renal function (Ashhab et al. 2001; Liu et al. 2011). These biomarkers remaining unchanged after long-term administration indicate that tsothel does not cause obvious early-phase renal injury.

Numerous *in vivo* and *in vitro* studies have suggested that oxidative damage is a principal underlying action of mercury-induced nephrotoxicity (Woods et al. 1990; Lund et al. 1993; Zalups 2000). Both SOD and MDA are quite important typical indexes in the oxidative system. SOD is a metalloenzyme that plays an important role in maintaining the balance between the oxidation and antioxidation systems. MDA is a terminal product of polyunsaturated fatty acid peroxidation and is often used as a marker of oxidative damage (Wei et al. 2010). In this research, it can be seen that tsothel did not have a significant effect on SOD activity or MDA concentration throughout the experiment, which indicates that the oxidative system in the kidney was not significantly damaged after long-term and large-scale exposure to tsothel; that is, the kidney has not yet suffered oxidative injury.

As previously mentioned Kim-1, a potentially valuable nephrotoxicity biomarker, can rapidly, sensitively and specifically reflect the early-phase injury in various kidney diseases. In addition, inorganic mercury has high affinity for sulfhydryl (SH) groups. MT, which is involved in the homeostasis of essential metals, is a low-molecular-weight, cysteine-rich metal-binding protein induced by metals and many other factors (Chan et al. 2002; Dabrio et al. 2002). Most importantly, MT is considered an important protective factor against renal toxicity caused by inorganic mercury and may play a major role in the retention of mercury in the kidney (Satoh et al. 1997; Tanaka-Kagawa et al. 1998). Additionally, GSH-Px is an antioxidative enzyme that constitutes approximately 90% of the intracellular SH groups (Gegg et al. 2003; Jurczuk et al. 2006), and it may combine with mercuric ions directly and reduce their levels in organisms. As one of the most versatile and pervasive mercury-binding peptides of γ-glutamylcysteinylglycine, GSH plays an important role in mercury transport, storage and distribution (de Ceaurriz et al. 1994). Endogenous GSH has a specific role in protecting the body from Hg toxicity due to its function as a carrier of Hg and its antioxidant properties. GSH binds with Hg and forms a complex that prevents Hg from binding to cellular proteins and subsequently causing damage to both enzymes and tissue (Kromidas et al. 1990).

In our study, after 90 days of administration, the Kim-1 and MT contents in the 33.35 mg/kg tsothel group increased, consistent with previous studies (Bonventre 2008; Lu et al. 2011; Shi et al. 2011). Based on the recuperation of Kim-1 and MT after another 90 days of administration, it was speculated that the increase in Kim-1 and MT contents was a response to increased stress, as the stress reaction and detoxification of the body occur when contacted the exogenous harmful substances for the first time. Furthermore, the GSH-Px activity in the 66.70 mg/kg tsothel group was significantly enhanced after 90 days of administration, and the GSH content in each tsothel group obviously increased after 180 days of administration. Nevertheless, the elevated GSH-Px activity and GSH content recovered and showed no difference from those in the control group. Theoretically, when kidney oxidative damage is induced by Hg, GSH-Px activity and GSH content will decrease or completely disappear due to the depletion of SH groups (Mahboob et al. 2001; Sener et al. 2003; Carocci et al. 2014). Therefore, our preliminary speculation was that the increase in GSH-Px activity and GSH content was a compensatory increase as tsothel induced stress injury by up-regulating the contents of Kim-1 and MT. Afterwards, with the extension of the administration period, the body had already developed a physical tolerance to the exogenous substance, namely, tsothel, and then the Kim-1 and MT contents and GSH-Px activity recovered gradually. In addition, the GSH content obviously increased later than the Kim-1 and MT contents and the GSH-Px activity in each tsothel group and was still high even after 30 days of drug withdrawal in the 33.35 mg/kg tsothel group. We suspect that the GSH sensitivity to tsothel, which may be weaker than that of the other three markers mentioned above, was responsible for its increase until 180 days of administration. In addition, another reason may be that the amount generated resulting in the change in GSH depends on the accumulation of tsothel. After 30 days of drug withdrawal, the GSH content was still high because its decreasing duration may be related to the dosage and contact time of tsothel. In this case, although the related biomarkers mentioned above showed abnormality after long-term administration, we speculated that the kidney still did not actually suffer oxidative damage due to the stress reaction and enzymatic detoxification system in the body. Theoretically, if the kidney suffers oxidative damage, GSH and GSH-Px should decrease. However, in this study, the amount of these three biomarkers increased, which indicates, from another perspective, that the kidney does not suffer oxidative injury. This result is in line with the SOD activity and MDA content results above.

The expression of some genes appears to be sensitive biomarkers for kidney injury. As previously mentioned, Kim-1 has proven to be an outstanding indicator of renal tubule injury in rats (Bonventre 2008; Prozialeck et al. 2009). MT-1 plays an important role in arsenic and mercury detoxification and thus is widely used as a biomarker for metal-induced toxicity (Klaassen et al. 1999). It is well known that there is a set of detoxifying enzyme systems in the body for endogenous and exogenous chemical substance, especially toxicants. Glutathione *S*-transferase (GST) isozyme, an important constituent of this enzyme system, mainly catalyzes the degradation of various chemical substances (e.g. drugs, chemotherapeutic agents, carcinogen). The covalent binding between some metabolites and the SH groups of GSH can protect some DNA and protein from injury (Veal et al. 2002; Lee et al. 2007). In particular, GST-Pi is a major isozyme in the GST family (Ramachandran et al. 2000). A remarkable result of this study is that the gene expression of GST-Pi was remarkably increased after 180 days of administration but was still high after 30 days of drug withdrawal. All results preliminarily indicate that the Hg that entered into the body may stimulate the detoxification system and then result in a stress reaction during administration. Thus, the gene expression levels of Kim-1, MT-1 and GST-Pi were increased through the induction of Hg, which then promoted the function of the detoxification system. This result indicates that tsothel has a certain potential renal injury trend. Moreover, it is predicted that one of the reasons for the high gene expression of GST-Pi may be its high sensitivity to toxicants and relatively long reversible recovery duration. Most interestingly, the change trend of GST-Pi gene expression is basically consistent with the change trend of GSH content in the experiments mentioned above, which further demonstrates that the detoxifying enzyme system is one of the key factors in the detoxification of tsothel-induced damage.

The H&E staining results indicated that the renal cell morphology was not damaged after long-term and large-scale exposure to tsothel; that is, there is not yet any pathological change in the kidney. Immunohistochemical staining initially indicated that tsothel may reduce the proliferation activity of the renal cells to some extent and that its mechanism may be related to the inhibition of Ki67 expression. Ki67 is a proliferating cell nuclear antigen (PCNA) related to the cell cycle. Its expression level reflects the degree of cell proliferation, that is, the more positive expression, the more active the cell proliferation. Therefore, it can be applied to identify the cells that are growing and to evaluate cell proliferation. Although its mechanism of cell cycle adjustment is still not clear, it is still regarded as a promising biomarker of cell proliferation (Endl and Gerdes 2000; Wong et al. 2006). Therefore, our study utilized these characteristics to observe the proliferation of renal cells.

The cell cycle is an orderly process that contains the early phase of DNA synthesis (G1), DNA synthesis (S), the later phase of DNA synthesis (G2) and the mitotic phase (M). Cell apoptosis is a process of regulating cellular suicide, and it closely associates with body development, the stable tissue environment in multicellular organisms, and the physiopathological environment (Herman-Antosiewicz and Singh 2004; Malumbres and Barbacid 2007). In our experiments, it can be seen that in the G0/G1 phase, S phase and G2/M phase, the apoptosis indicators in the renal cells in all tsothel groups were not markedly altered with time. Notably, the cell cycle and apoptosis actually reflect a process of cell proliferation. Based on the Ki67 expression result described above, tsothel has some impact on cell proliferation activity, but its effect on the proliferation process is not substantially obvious.

Additionally, the results presented in this paper clearly demonstrate that the toxicity potential of tsothel was much less than that of common mercurials (MeHg and HgCl_2_), which is similar to the results of recent studies comparing the toxicity of mercury-containing traditional medicines with that of common mercurials (Shi et al. 2011; Lu et al. 2011; Wang et al. 2013). As mentioned above, tsothel contains 54% mercuric sulphide, numerous herbs, animal medicines and some mineral substances. Additionally, tsothel is insoluble and thus may be poorly absorbed from the gastrointestinal tract. Thus, it is not surprising that the toxicological potential of tsothel is quite different from that of common mercurials. In the present study, the renal Hg accumulation in the tsothel groups was some thousands-fold higher than that in the control group, but its nephrotoxicity potential seemed to be tolerable and reversible. It has been speculated that ‘soluble Hg’ or ‘free Hg’ contributes to the potential nephrotoxicity of HgS rather than the total Hg content (Shi et al. 2011). Therefore, the renal Hg accumulation in our present study was always at a high level, but the mercuric components contributing to the potential nephrotoxicity of tsothel were probably just small amounts that were not enough to induce obvious and irreversible nephrotoxicity. As several studies have shown, mercuric polysulphides are the major dissolved components released from HgS, and their behaviour is quite different from that of HgCl_2_ in binding serum protein (Zhou et al. 2010). Furthermore, the release of Hg (++) from HgS (cinnabar) is more difficult than that from HgCl_2_ (Lu et al. 2011). Elemental mercurials, inorganic mercurials and organic mercurials must be distinguished when discussing their toxicity (Klaassen 2006), and the total mercury content alone is inappropriate for the safety evaluation of tsothel and HgS-containing traditional medicines (Zalups and Lash 1994). This prompts us to note that the renal toxicity of mercurial depends not only on the total Hg content but also on the chemical forms and physical status (Zalups and Lash 1994; Shi et al. 2011; Lu et al. 2011). Therefore, the use of the total Hg content alone to evaluate the safety of HgS-containing traditional medicines is not scientifically sound, and chemical forms of the metal and their disposition should be taken into consideration in risk assessment. Moreover, it is also important to avoid overdose, long-term use, and improper processing of HgS-containing traditional medicines to reduce potential renal injury.

## Conclusions

In summary, all the data presented in this report demonstrated that the traditional Tibetan medicine tsothel generally had no significant renal toxicity but did exert stress-related effects. The formulation method and chemotype could play a role in the reduced toxicity potential of tsothel compared to common mercurials. Nevertheless, tsothel leads to the accumulation of Hg in the kidney even after long-term drug withdrawal. More importantly, tsothel can cause potentially reversible kidney injury. The mechanism, at least in part, is probably closely associated with the stress response and detoxification caused by detoxifying enzyme systems, which was a key point in our study. Specifically, this assumption is supported by the relative expression levels of Kim-1, MT-1 and GST-Pi mRNA. Thus, avoiding overdose or long-term use could reduce potential renal injury. Furthermore, there is also a need to carry on an in-depth study on the toxicity and mechanism of the Hg residue after a longer drug withdrawal period. We hope that our results will stimulate more interest in studies on the biosafety of tsothel.
